# Diabetes Type 2 in Neurologically Impaired Children and Adolescents Without Obesity: A New Emerging Entity?

**DOI:** 10.3389/fneur.2019.00947

**Published:** 2019-08-29

**Authors:** Valeria Calcaterra, Hellas Cena, Annalisa De Silvestri, Vincenza Girgenti, Denisia Bommarito, Gloria Pelizzo

**Affiliations:** ^1^Pediatric and Adolescence Unit, Department of Internal Medicine, University of Pavia, Pavia, Italy; ^2^Pediatric Unit, Department of the Mother and Child Health, Fondazione IRCCS Policlinico San Matteo, Pavia, Italy; ^3^Laboratory of Dietetics and Clinical Nutrition, Department of Public Health, Experimental and Forensic Medicine, University of Pavia, Pavia, Italy; ^4^Unit of Internal Medicine and Endocrinology, Clinical Nutrition and Dietetics Service, ICS Maugeri IRCCS, Pavia, Italy; ^5^Biometry & Clinical Epidemiology, Scientific Direction, Fondazione IRCCS Policlinico San Matteo, Pavia, Italy; ^6^Pediatric Surgery Department, Children's Hospital “G. Di Cristina”, ARNAS Civico-di Cristina-Benfratelli, Palermo, Italy

**Keywords:** type 2 diabetes, children, adolescents, neurologically impaired, disability

## Abstract

**Background:** Insulin resistance (IR) plays a key role in the pathogenesis of type 2 diabetes (T2D). In neurologically impaired (NI) children unfavorable cardio-metabolic risk profile with high prevalence of IR has been reported. We evaluated the prevalence of T2D in NI children and adolescents, in order to define if a dedicated glucose monitoring may be recommended in these subjects.

**Methods:** We retrospectively evaluated 63 patients (11.4 ± 4.0 years) with severe disabilities. Auxological parameters were recorded. Metabolic blood assays included fasting blood glucose (FBG), fasting insulin, triglycerides (TG). IR was detected with the homeostasis model assessment for insulin resistance (HOMA-IR > 97.5th percentile for age and sex) and triglyceride-glucose index (TyG index > 7.88). Elevated FBG was defined with values >100 mg/dl. T2D was defined according to American Diabetes Association criteria.

**Results:** Impaired insulin sensitivity, pathological TyG index and elevated FBG were observed, respectively, in 41.3, 63.5, and 11.1% patients. T2D was diagnosed in 3.2% asymptomatic patients. The prevalence of diabetes was higher in pre-pubertal compared to pubertal subjects (*p* = 0.03).

**Conclusions:** T2D in NI children and adolescents without obesity could represent a new emerging entity. IR and/or surrogate markers of IR index may be useful for the primary screening of this at-risk disabled population so as to prevent diabetes.

## Introduction

Youth type 2 diabetes (T2D) is increasing, linked with obesity and declining physical activity in high-risk population (1). In the United States, the prevalence of T2D in 2009 among adolescents aged 10 through 19 years was 0.46 per 1,000 or 0.046%, with highest prevalence in American Indians, followed by black, Hispanic, and Asian Pacific Islander youths, with lowest prevalence in white ones (1–3). Studies in Europe indicate that T2D remains rare in largely white populations (4–8).

Insulin resistance (IR) plays a key role in the pathogenesis of T2D (1). Previously, we described that neurologically impaired (NI) children showed an unfavorable cardio-metabolic risk profile with a high prevalence of IR which was not related to BMI (9–11).

In this brief report, we evaluated the prevalence of T2D in NI children and adolescents without obesity, in order to describe if a dedicated glucose profile monitoring may be recommended in this “fragile” population.

## Patients and Methods

### Patients

We retrospectively evaluated 63 caucasian patients (35 males/28 females, mean age 11.4 ± 4.0 years, range 1.9–19.1 yr) with severe disabilities (cerebral palsy 34.9%, epileptic encephalopathy 28.6%, severe psychomotor developmental delay in dysmorphic syndromes 36.5%). All patients presented with neuromotor damage that could alter their ability to self-feed and were bedridden.

All subjects had been previously scheduled for surgical treatment (gastrostomy/jejunostomy tube positioning, anti-reflux surgical treatment) and/or management of nutritional support devices.

Enteral feeding was adopted in 58/63 participants (bolus 82.7%, continuous pump feeding 17.3%) and oral feeding in 5/63 subjects (8%). Anticonvulsive drugs (at least two of the following: phenobarbital, valproic acid, phentoyn, lamotrigine, topiramate, carbamazepine, and clonazepam) were reported in 56/63 (88.8%) of the whole sample; other therapies were recorded in 5 patients (in 3 anti-hypertensive drugs, in 1 growth hormone therapy, in 1 L-thyroxine).

In all subjects physical activity was conducted for <60 min/week.

The study was performed according to the Declaration of Helsinki and with the approval of the Institutional Review Board. Parents and/or legal guardian, after receiving information about the study, gave their written consent.

### Methods

#### Clinical and Anthropometric Parameters

Physical examination of the subjects included anthropometric parameters as well as pubertal stage evaluation according to Marshall and Tanner (12, 13) (pre-pubertal characteristics corresponding to Tanner stage 1).

Weight, height, waist circumference (WC) were measured as previously reported (11) and consequently BMI and waist to height ratio (WHtR) were calculated.

#### Metabolic Parameters

Metabolic blood assays included fasting blood glucose (FBG), insulin, triglycerides (TG). Insulin resistance was calculated with the homeostasis model assessment for insulin resistance (HOMA-IR) (14). Triglyceride-glucose index (TyG index) was also evaluated using the formula (ln[fasting triglycerides (mg/dl) × fasting plasma glucose (mg/dl)/2]) (15), as a surrogate marker of IR and predictor of diabetes.

Elevated FBG was defined with values exceeding 100 mg/dl and impaired insulin sensitivity (ISI) with a HOMA-IR exceeding the 97.5th percentile for age and sex (16).

According to Vieira Ribeiro, TyG index was considered pathological with a cut-off exceeding 7.88.

T2D was defined as FBG ≥ 126 mg/dL and/or 2 h plasma glucose (PG) ≥ 200 mg/dL and/or random PG ≥ 200 mg/dL (in patients with continuous pump feeding only random PG ≥ 200 mg/dL was considered) plus symptoms of diabetes (given the feeding difficulties, of these children, only polyurea was considered) and HbA1c ≥ 6.5% (17).

In diabetic patients the presence of islet autoimmunity [glutamic acid decarboxylase (GAD), islet antigen type 2 (IA2), anti-insulin (IAA), and zinc transporter 8 (ZnT8) autoantibodies] were excluded.

The waist to height ratio (WHtR) was then calculated with the standard formula and a cut-off of 0.5 was used to differentiate low from high WHtR (18).

Treatment following diabetes diagnosis was recorded.

### Statistical Analysis

The Shapiro-Wilk test was used to assess the normal distribution of quantitative variables. When quantitative variables were normally distributed, the results were expressed as the mean value and standard deviation (SD), otherwise median and interquartile range (IQR; 25th−75th percentile) were reported. Qualitative variables were summarized as counts and percentages. Associations between sex or pubertal stage and continuous variables were tried through *t*-test or non parametric Mann-Whitney test for the former and oneway analysis of variance followed by Scheffe corrected 2 × 2 *post hoc* test for the latter; chi square test was used for qualitative variables. A *p* < 0.05 was considered statistically significant. Multivariate (including sex and pubertal stage as independent variables) linear or logistic regression models were fitted.

## Results

In Table 1, the clinical features and metabolic parameters of the patients are reported.

**Table 1:** Clinical and metabolic feature of the patients.

Sex (M/F)	35/28
Age (years)	11.4 ± 4.0
Pubertal stages	
0 (Tanner stage 1) 1 (Tanner stage 2–3) 2 (Tanner stage 4–5)	15 20 28
BMI (kg/m^2^)	12.01 ± 5.91
BMI-SDS (kg/m^2^)	−2.26 ± 2.86
Waist circumference (cm)	67.9 ± 15.2
Waist-to-height ratio	0.3 ± 0.2
Fasting blood glucose (mg/dl)	88.3 ± 9.1
Fasting insulin (micro IU/ml)	19.9 ± 23.9
Triglycerides (mg/dl)	108.1 ± 8.5
HOMA-IR	3.5 ± 0.6
Triglyceride-glucose index	8.1 ± 0.75

The average HOMA-IR was 3.5 ± 0.6, with higher levels in pubertal compared to pre-pubertal patients (*p* = 0.03) and without significant difference between sex (*p* = 0.3). Insulin resistance was detected in 26/63 (41.3%) subjects, with a similar prevalence in males and females (*p* = 0.7) and in pre-pubertal and pubertal subjects (*p* = 0.1).

The mean values of TyG index observed in the sample was 8.1 ± 0.75, which is higher in pubertal compared to pre-pubertal patients (*p* = 0.03) and did not differ between genders (*p* = 0.6). Pathological TyG index was noted in 40/63 patients (63.5%), without significant difference according to sex (*p* = 0.3) and pubertal status (*p* = 0.6).

No significant correlation between HOMA-IR and TyG was found (*r* = 0.17 *p* = 0.20).

Pathological FBG was detected in 7/63 children (11.1%), without difference between sex (*p* = 0.3 and pubertal stage (*p* = 0.09).

T2D was diagnosed in 2/63 patients (3.2%; 1 male and female with dysmorphic syndromes in which inborn errors of metabolism and mitochondrial disease were excluded) at the age of 4 and 8 years, respectively. Both patients were asymptomatic and diabetes was incidentally detected during a routine checkup. Clinical and biochemical data and treatment following diagnosis was reported in Table 2. In both patients, IR or surrogate markers of IR were detected.

**Table 2:** Clinical and metabolic feature of the 2 patients with diabetes.

	**Patient 1**	**Patient 2**
Sex	Male	Female
Age	4	8
Family history for diabetes	Absent	Absent
Neurological disorders	Lissencephaly with cerebellar dysplasia	Corpo callosum hypoplasia, delayed myelination, simplified gyration
Concomitant pathologies	None	Biatrial enlargement
Treatment	Anticonvulsive drugs	Growth hormone
Ketoacidosis at diagnosis	Absent	Absent
Symptoms recorded at diagnosis	None	None
Glycemia at the diagnosis	355	490
HBA1c	7.8	9.5
Waist circumference (cm)	50 (<50° centile)	48 (<50° centile)
Waist-to-height ratio	0.45	0.44
BMI (kg/m^2^)	14.8	12.6
BMI *z*-score	1.5	−1.6
HOMA-IR	10.6	1.6
Triglyceride-glucose index	9.58	9.83
c-peptide	Not available	1.6
**Autoimmune markers**		
Islet cell autoantibodies Autoantibodies to GAD (GAD65) Autoantibodies to the tyrosine phosphatases IA-2 Autoantibodies to zinc transporter 8 (ZnT8)	Not available Negative Negative Not available	Negative Negative Negative Negative
HLA risk haplotypes	Not available	Absent
Therapy following diagnosis	Metformin	Insulin

The prevalence of diabetes was higher in pre-pubertal compared to pubertal subjects (*p* = 0.03), similarly in males and females (*p* = 0.8).

No association between metabolic alterations and type of nutritional support (*p* = 0.1) or drug exposure was noted (*p* = 0.9).

## Discussion

Individuals with neurological impairment are at increased risk for frailty and chronic disease due to factors experienced throughout lifespan, such as excessive sedentary behaviors and malnutrition (19). Unfavorable cardio-metabolic profile and high allostatic load have been previously reported in early childhood (9–11). However, little is known about diabetes in disabled population.

This is the first study to report a certain prevalence of diabetes mellitus (3.2%) in the NI pediatric population. We demonstrated that ISI and pathological surrogate markers of insulin resistance index are common in children and adolescents with NI and showed that a high prevalence of diabetes is also relevant in pre-pubertal subjects.

Glucose homeostasis is maintained by a delicate coupling of insulin secretion, from pancreatic β-cells, with insulin sensitivity (skeletal muscle, adipose tissue, and hepatic) (1). When insulin sensitivity declines, insulin secretion must increase to maintain glucose tolerance and finally when β-cells are no longer able to secrete sufficient insulin to overcome insulin resistance, IGT ensues progressing to T2D (1).

Overweight and obesity are major contributors to the development of T2D, which represents the 1% of diabetes subtype in the italian pediatric population (20). There are few data on T2D prevalence in children without obesity and in this population diabetes is described as a major complication of post-transplant immunosuppressive therapy for kidney (21), liver (22), or hematological diseases (23) with a broad range (1.8–13%) depending of the treatment process (21–23).

We showed that the development of T2D and IR may also be observed in NI patients without obesity, not on immunosuppressive drugs, with no difference between sex. As previously reported, IR in these disabled patients was not correlated to BMI nor to energy intake (11). Negative regulation of insulin signaling could be viewed as a physiologic “adaptive mechanism for human survival” that is activated whenever the organism needs to switch from an anabolic to a catabolic or “insulin resistance” state, such as undernutrition, and to mobilize energy to support vital metabolic processes (9–11, 24–26). The restricted physical activity (27) may also play a crucial role on insulin resistance; it causes a rapid loss of lean mass, which is associated with a decline in basal metabolic rate and increased whole body and regional adiposity leading to insulin signaling interference (10). Additionally, chronic stress also tends to alter the anabolic/catabolic hormonal balance and may be involved in increased cortisol levels and insulin resistance (28). Finally, long-term therapy, such as anticonvulsive drugs and hormonal therapy may be associated with several metabolic abnormalities and their effects on insulin resistance and diabetes should be considered (9–11).

Glucose and lipid metabolism are linked to each other in many ways (29). Hypertriglyceridemia is the characteristic dyslipidemia in subjects with IR (29). Although no definitive explanation is still available for the correlation between hypertriglyceridemia and IR, it has been reported that elevated TG levels interfere with glucose metabolism in muscles, a finding consistent with the hypothesis that TG elevation in serum and tissue is related to decreased insulin sensitivity (29–31). Markers of insulin action based on lipids may help identify subjects with IR (32–36). The TyG index which is a surrogate marker of IR, is known to be associated with metabolic parameters and CVD and it had some prognostic value to predict T2D also in normoglycemic patients and normal-weight patients (32–34, 36). In our report a higher percentage of patients without obesity, with significant difference between sexes, showed pathological TyG index, in accordance with the presence of IR. Considering the unfavorable cardio-metabolic risk profile of this disabled population (9–11), this marker may be useful for predicting T2D in clinical practice and may provide a feasible alternative to the expensive and invasive gold standard test for IR.

We observed a lack of correlation between HOMA-IR and TyG index; this result could be due to the fact that the HOMA-IR does not change at the same time as the TyG index. Youth T2D is associated with insulin resistance, together with progressive deterioration in cell function and relative insulin deficiency in the absence of diabetes-related immune markers (1). Therefore HOMA-IR may not be altered when insulin reserves are running out. Further longitudinal studies are mandatory to clarify the natural history of ISI and β-cell Function in neurologically impaired youth.

We must acknowledge that the study has some limitations. The sample size was too small to define a prevalence of diabetes mellitus, moreover due to the difficulties in enrolling and studying neuro-cognitively disabled children, this metabolic aspect is interesting to consider for disabled young patients monitoring. The gold standard method for the determination of insulin sensitivity is the hyperinsulinemic euglycemic glucose clamp, moreover it is impractical in these young disabled people so we used HOMA-IR and TyG index because. Finally, several factors, such as inadequate nutritional status, low physical activity level, polypharmacotherapy, may synergically affect metabolic profile, however the effects of any single event was not measured. Additionally, a genetic component of diabetes is not excluded.

In conclusion, T2D in NI children and adolescents could represent a new emerging entity in subjects without obesity. Insulin resistance and/or surrogate marker of insulin resistance index may be useful for the early screening of these at-risk disabled populations in order to prevent diabetes.

## Data Availability

All datasets generated for this study are included in the manuscript.

## Author Contributions

VC and HC designed experiments, wrote, and supervised the manuscript. VG and DB carried out experiments. AD performed statistical analysis. GP provided patient samples, wrote, and supervised the manuscript. All authors have read and approved the manuscript.

### Conflict of Interest Statement

The authors declare that the research was conducted in the absence of any commercial or financial relationships that could be construed as a potential conflict of interest.
